# Age-related differences and common pathways of lymphocyte subsets in sepsis: a comparative review of elderly and pediatric patients

**DOI:** 10.1038/s41419-026-08773-3

**Published:** 2026-05-05

**Authors:** Xianwen Wang, Qihang Huang, Zhihong Zuo, Zhanwen Wang, Lina Zhang, Zhaoxin Qian

**Affiliations:** 1 Department of Critical Care Medicine, Hunan Provincial Clinical Research Center for Critical Care Medicine, Xiangya Hospital, Central South University, Changsha, Hunan China; 2 National Clinical Research Center for Geriatric Disorders, Xiangya Hospital, Central South University, Changsha, Hunan China

**Keywords:** Infection, Lymphocytes, Immunotherapy

## Abstract

Sepsis disproportionately affects older adults and children, two immunologically vulnerable extremes of age. Yet sepsis is superimposed on distinct baselines—immunosenescence in the elderly and immune immaturity in neonates and young children—leading to different pathways toward immune failure. This comparative narrative review synthesizes clinical and experimental evidence on age-specific and shared alterations in lymphocyte subsets in sepsis, including lymphopenia; CD4^+^ and CD8^+^ T cell activation, apoptosis, and exhaustion; B cell depletion and impaired antibody production; NK cell cytotoxic defects; and dynamic regulatory circuits such as Tregs. Recognizing that early organ injury is initiated and amplified primarily by innate immune programs, we frame lymphocyte injury largely as a downstream cost of the acute host-response milieu that can become rate-limiting for immune recovery, secondary infections, and late mortality. We highlight convergent phenotypes linked to secondary infections and late mortality, while emphasizing differences in kinetics, mechanisms, and recovery potential. We propose an age-stratified approach to serial immune monitoring and biomarker-enriched trial design to guide immunoadjuvant therapies and avoid one-size-fits-all immunomodulation. Clarifying these trajectories may improve risk stratification and outcomes across the lifespan.

## Facts

- Early sepsis is driven mainly by innate amplification; lymphocyte loss is often collateral damage, but persistent lymphopenia can become rate-limiting for recovery. The same phenotype may mean reconstitution failure (older adults) versus developmental derailment (children).
- The best longitudinal, age-calibrated biomarker set to define “sustained immunosuppression” remains unsettled—how to integrate ALC kinetics, mHLA-DR recovery, checkpoint/exhaustion markers, and functional assays (especially in pediatrics) lacks standard thresholds.
- Checkpoint/exhaustion-like signals are not uniformly pathogenic across ages. In pediatrics, phenotypic similarity (e.g., PD-1/PD-L1, Tim-3, LAG-3) may reflect developmental restraint rather than adult-like dysfunction; interpretation requires time anchoring and functional confirmation.
- B cell and NK cell defects are common but under-measured; whether correcting humoral memory/antibody quality or restoring cytotoxic competence reduces secondary infections and late mortality—and whether effects are age-specific—is unclear.
- Many immunotherapy trials likely failed due to syndrome-based enrollment rather than immune trajectory. Priority: age-stratified, biomarker-enriched, time-sensitive trials that restore host defense while limiting rebound hyperinflammation.

## Introduction

Sepsis is life-threatening organ dysfunction caused by a dysregulated host response to infection and remains a major global public health challenge. Global Burden of Disease analyses estimated 48.9 million incident cases and 11.0 million sepsis-related deaths worldwide in 2017, accounting for almost one in five deaths globally [1]. The burden is concentrated in low- and middle-income countries and disproportionately affects the extremes of age [1, 2]. This review focuses on these extremes: pediatric/neonatal sepsis remains common and lethal, particularly in the neonatal period [3, 4], while adults ≥65 years constitute an expanding share of severe sepsis and septic shock with higher organ failure rates and mortality than younger adults [5–7]. Together, these age groups represent both the highest-risk hosts and the most divergent immune baselines.

Sepsis is not a single “hyperinflammatory” state. Early inflammatory surges can coexist with compensatory anti-inflammatory programs and sustained immune dysfunction [8, 9]. Clinically, the key shift is from acute injury to impaired host defense. This immune dysfunction includes lymphocyte apoptosis, exhaustion of surviving T cells, impaired antigen presentation, and expansion of regulatory/suppressor circuits [8–11]. Tissue studies in fatal sepsis show extensive CD4⁺ and CD8⁺ T cell and B cell apoptosis with increased inhibitory receptor expression (e.g., PD-1, CTLA-4), consistent with profound adaptive immunosuppression (often termed immunoparalysis when severe and persistent) [12]. Multi-omics analyses further link checkpoint upregulation, transcriptional rewiring, and metabolic reprogramming of T cells to adverse outcomes [13]. Because the acute septic response unfolds over hours to days, early organ injury is initiated and amplified primarily by innate immune sensing and effector pathways; lymphocyte depletion and dysfunction are therefore often downstream consequences of the early innate/stress milieu rather than primary initiators. Once established, adaptive immune injury can become rate-limiting for recovery, shaping secondary infections and late deterioration and motivating serial immune monitoring and time-sensitive, biomarker-enriched immunoadjuvant trials [8–11, 13].

Throughout this review, we use immune dysfunction as an umbrella term for sepsis-associated perturbations across immune compartments and functions. We use immunosuppression to denote a clinically relevant reduction in antimicrobial competence (e.g., impaired antigen presentation, lymphocyte depletion, reduced effector function) that increases vulnerability to secondary/opportunistic infections. We reserve immunoparalysis for a more severe and persistent state of immunosuppression that is operationalized by longitudinal persistence (failure to rebound) and/or functional impairment despite initial stabilization and source control, rather than by a single early “snapshot” biomarker.

Age fundamentally resets the immune “starting line,” because immunity is continuously remodeled across the lifespan—from immune ontogeny to intrinsic aging processes [14]. In older adults, immunosenescence includes chronic low-grade inflammation (“inflammaging”), contraction of the naïve T cell pool, accumulation of memory/terminally differentiated subsets, impaired B cell function, reduced NK cell cytotoxicity, and myeloid skewing toward a pro-inflammatory yet functionally compromised phenotype [15–17]; these constraints track with worse sepsis outcomes and a stronger propensity toward sepsis-induced immunosuppression in the elderly [7, 17]. In neonates and young children, the immune system is “immature” rather than “aged,” with distinct innate signaling/cytokine outputs and an adaptive compartment dominated by naïve lymphocytes with limited antigen experience and incompletely established memory/regulatory networks [14, 18, 19]. This baseline confers vulnerability but also greater plasticity in recovery trajectories [18–20]. Consequently, similar bedside phenotypes can reflect different biology: impaired reconstitution on a senescent baseline versus disruption of a developing immune program.

**Fig. 1:**
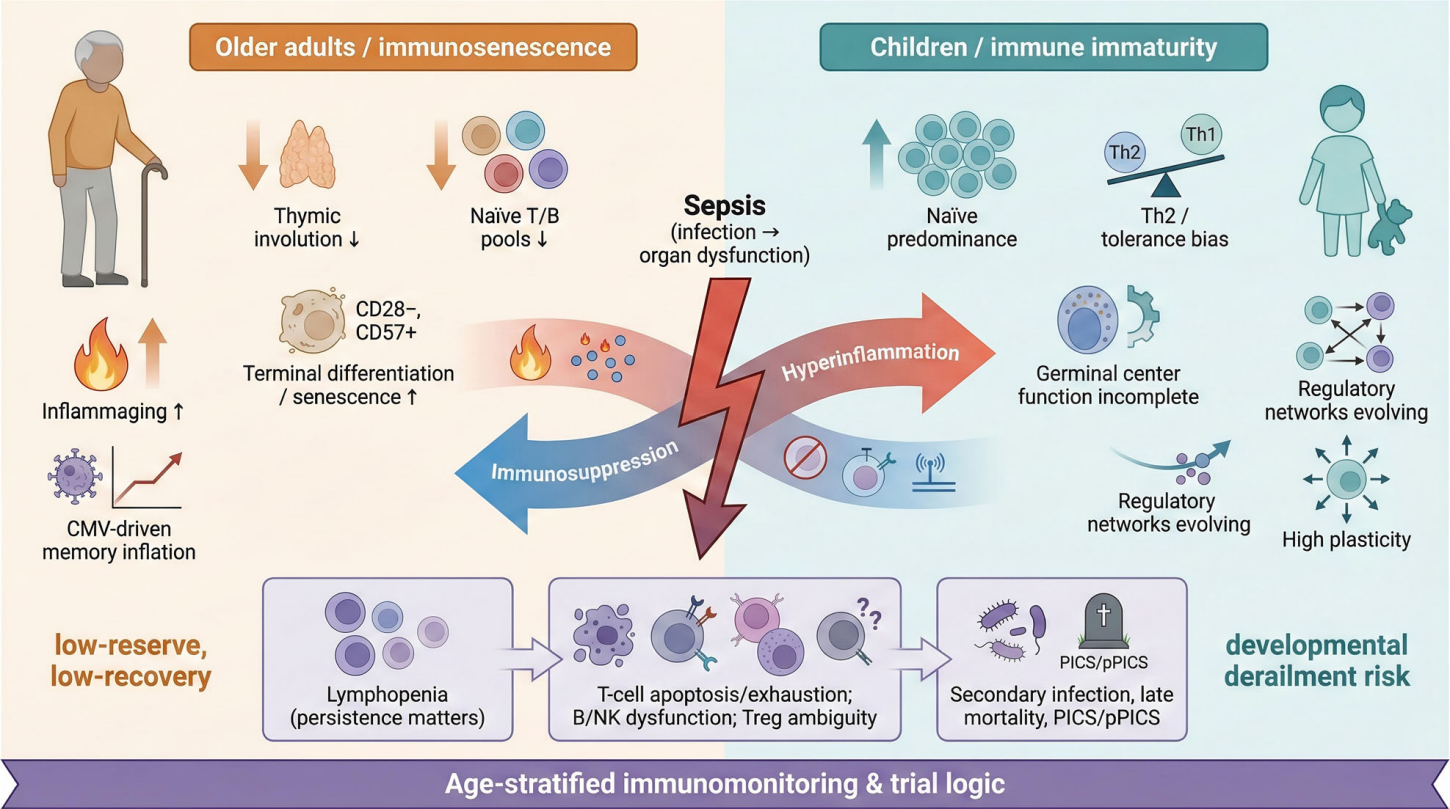
Age-conditioned immune “starting lines” shape sepsis-induced lymphocyte trajectories at the extremes of age. Older adults enter sepsis on a baseline of immunosenescence, characterized by reduced thymic output and naïve lymphocyte reserve, expansion of terminally differentiated/senescent phenotypes, and chronic low-grade inflammation, predisposing to a “low-reserve, low-recovery” trajectory. Children enter sepsis while immune circuits are still being assembled; naïve predominance, developmental polarization biases, and evolving humoral and regulatory networks confer plasticity but also vulnerability to “developmental derailment”. A septic insult can simultaneously trigger inflammatory injury and immunosuppression, converging on lymphopenia and multi-lineage functional impairment (T cell apoptosis/exhaustion, B cell and NK cell dysfunction, and context-dependent regulatory dominance). Despite shared downstream phenotypes, the upstream constraints differ by age, motivating age-stratified immunomonitoring and endotype-aware trial design to reduce secondary infections and late complications. Clinical take-home: baseline “starting lines” determine how to interpret lymphocyte trajectories and what “recovery” can plausibly look like; therefore, immune monitoring and trial logic must be age-stratified.

**Fig. 2:**
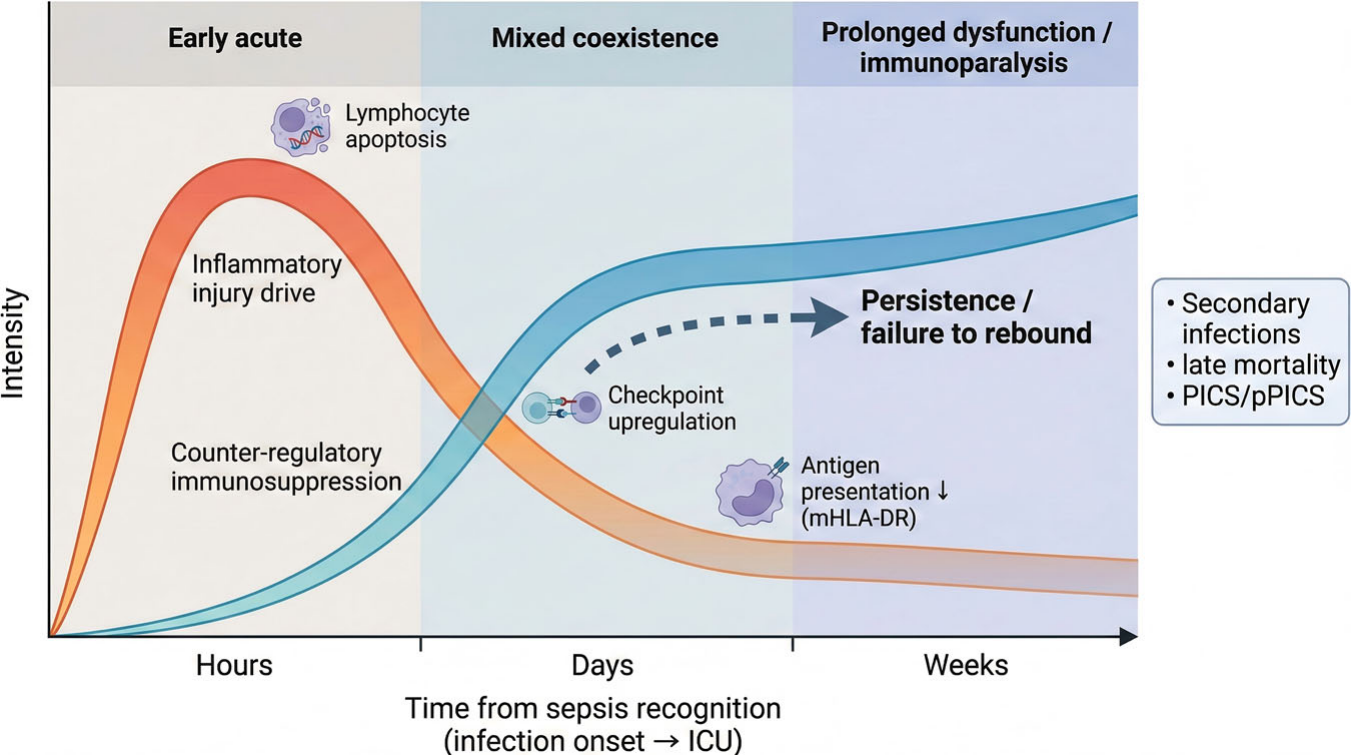
Immune trajectories in sepsis: coexistence of inflammatory injury and immunosuppression and the importance of kinetics. Sepsis is a dynamic disturbance of immune homeostasis in which inflammatory injury and immunosuppression can coexist rather than unfold as clean sequential phases. Lymphocyte apoptosis, inhibitory checkpoint upregulation, and impaired antigen presentation contribute to an immunosuppressive trajectory that can persist beyond early resuscitation. Clinically, the persistence of lymphopenia (failure to rebound), together with delayed recovery of antigen-presenting capacity, provides a pragmatic signal of sustained immune dysfunction associated with secondary infections and late adverse outcomes. Clinical take-home: interpret lymphocyte- and antigen-presentation–related biomarkers by kinetics; persistence beyond early resuscitation (failure to rebound) is the most actionable signal for risk stratification and trial enrollment.

Despite advances in sepsis immunobiology, direct, structured comparisons of lymphocyte dysfunction between elderly and pediatric sepsis remain limited [3–6, 17–20]. Existing evidence suggests shared endpoints—lymphopenia, T cell apoptosis/exhaustion, B cell dysfunction, NK cell defects, and regulatory expansion—while kinetics, drivers, and recovery ceilings diverge across ages [3–7, 17–20]. Our central message is that identical bedside phenotypes (e.g., persistent lymphopenia or secondary infections) can encode different immunobiological trajectories at the extremes of age—failed immune reconstitution on an immunosenescent baseline in older adults versus derailment of an actively developing immune program in children—so monitoring and intervention must be both age-stratified and trajectory-based. The remainder of the review applies this lens across lymphocyte subsets. Table 1 anchors baseline differences; Fig. 1 summarizes divergent recovery constraints; and Fig. 2 emphasizes persistence (failure to rebound) as the pragmatic clinical signal. Against this backdrop, we synthesize age-specific and shared alterations across major lymphocyte subsets and translate them into practical principles for age-stratified immune monitoring, risk re-stratification, and biomarker-enriched trial design in sepsis.

**Table 1:** Baseline immune landscape at the extremes of age relevant to sepsis-induced lymphocyte injury.

Immune domain	Older adults (immunosenescence baseline)	Neonates/children (immunoimmaturity baseline)	Sepsis implication (why this matters)
T cell supply & repertoire	Thymic involution → reduced de novo T cell output; contraction of naïve T cell pool; repertoire narrowing; memory inflation (often CMV-associated) [47–54]	Large naïve pool; limited antigen experience; developmental biases in effector programming [14, 18–20, 66–68]	Elderly: “low-reserve, low-recovery” once collapse occurs; Children: risk of derailing immune maturation trajectories rather than pure exhaustion [14, 46, 94–99]
Dominant T cell phenotypes	Expansion of terminally differentiated/senescent-like (e.g., CD28⁻, CD57⁺/KLRG1⁺) and exhausted features [46, 50, 51, 60]	Naïve predominance; Th2/tolerance-prone tendency early in life [66–70]	Same clinical phenotype (e.g., lymphopenia) can reflect different biology, requiring age-aware interpretation and trial logic [9, 10, 93–95]
Baseline inflammatory tone	Chronic low-grade inflammation (“inflammaging”) [15–17]	Regulatory networks still forming; maternal factors and microbiome shape tone [73–77]	Elderly may enter sepsis with higher inflammatory background yet weaker adaptive recovery; pediatrics has higher plasticity but greater variability by developmental stage
B cell compartment	Reduced B cell output/repertoire diversity; weaker responses to new antigens [55, 56]	Germinal center function still maturing; repertoire breadth expanding with exposures [71]	Elderly: impaired antibody quality and recall; pediatrics: sepsis may impair both acute defense and consolidation of durable memory [55, 56, 71]
NK cell biology	Counts may be preserved but cytotoxicity/function often reduced with age [57, 58, 65]	Phenotypically present but functionally reduced in newborns [72]	“Double hit” risk at both ends: innate cytotoxic containment weakens alongside adaptive dysfunction [57, 58, 62, 72]
Key baseline modifiers to record	CMV serostatus, frailty, comorbidity burden, polypharmacy [50–54, 116–118]	Gestational age, postnatal age, breastfeeding/maternal IgG exposure, vaccination history [73–77]	These modifiers meaningfully shift “normal ranges” and biomarker thresholds, especially in pediatrics [69, 80]

Baseline features summarize age-conditioned immune architecture present before sepsis onset (or at the earliest measurable time point), which constrains the depth, kinetics, and reversibility of sepsis-related lymphocyte injury. Immunosenescence and immune immaturity denote population-level patterns with substantial within-age heterogeneity; key modifiers (e.g., frailty/comorbidity burden and CMV serostatus in older adults; gestational/postnatal age and developmental stage in pediatrics) should be recorded when available. Comparisons primarily reflect peripheral blood and should be interpreted using explicit pediatric age bands. Clinical take-home: record baseline modifiers whenever feasible, because they shift “normal ranges” and the interpretation of persistence (failure to rebound), particularly in pediatrics.

*CMV* cytomegalovirus, *NK* natural killer, *TCR* T cell receptor, *BCR* B cell receptor, *ICU* intensive care unit.

### The landscape of lymphocyte alterations in sepsis: general paradigms

As defined by Sepsis-3, sepsis is life-threatening organ dysfunction driven by a dysregulated host response to infection [21]. Clinical deterioration reflects not only inflammatory injury but also collapse of antimicrobial competence, with high susceptibility to secondary/opportunistic infections and late mortality [9, 10, 22, 23]. This phenotype reflects coupled dysfunction across innate and adaptive immunity: an early, rapidly amplified myeloid-driven response can coexist with—or precipitate—collapse of immune competence. In this section, we focus on lymphocyte trajectories as accessible markers of immune injury and as plausible determinants of secondary infection risk and late outcomes, while explicitly recognizing that these adaptive changes are shaped and constrained by earlier innate events and host-response heterogeneity [9, 10, 12, 22–25]. Molecular endotyping reinforces that sepsis does not map to a single immune state; host-response signatures stratify distinct immune trajectories and outcomes [26, 27].

### Innate immunity sets the tempo in early sepsis

Sepsis begins with rapid innate immune detection and signal amplification through PRRs, followed by immediate cytokine release, complement activation, and coagulation–inflammation crosstalk that can drive early tissue injury [8, 9, 21]. Importantly, innate effector heterogeneity and immunoregulatory reprogramming emerge within the same time window: monocyte deactivation (e.g., reduced HLA-DR), neutrophil dysfunction (impaired chemotaxis and dysregulated NET formation), and suppressor-like myeloid phenotypes can coexist with hyperinflammation [8–10]. These early innate programs create the systemic milieu that both injures lymphocytes and constrains subsequent adaptive recovery, providing a mechanistic bridge between “innate-driven onset” and “adaptive-limited late host defense,” especially at the extremes of age.

### The widespread occurrence of lymphopenia

Lymphopenia is among the most accessible bedside footprints of adaptive immune injury in sepsis [24]. Prognosis is driven less by the first count than by kinetics: persistent lymphopenia tracks excess mortality [25]. Mechanistically, apoptosis is a dominant early driver, with marked depletion of B cells and CD4⁺ T cells documented in human sepsis [28]. This early apoptosis phenotype tracks with the intensity of the innate inflammatory and neuroendocrine stress response, including pro-inflammatory cytokines and death-receptor ligands, stress-hormone signaling, and acute immunometabolic collapse converging on mitochondrial dysfunction and programmed cell death. Framing lymphopenia as “collateral damage” from an innate-driven storm reconciles its early onset with slower antigen-specific adaptive kinetics while preserving its downstream relevance. Tissue-level studies in fatal sepsis further support profound, systemic immunosuppression [12]. Proof-of-mechanism immunorestoration exists: recombinant IL-7 can expand circulating lymphocytes and related immune readouts in septic shock, although outcome benefit remains unproven [29]. Viral reactivation provides a clinical correlate of immune collapse in critical illness, including cytomegalovirus reactivation and broader reactivation of latent viruses in sepsis cohorts [30, 31].

### Imbalance of T cell subsets: abnormal CD4^+^/CD8^+^ ratio and Th polarization shifts

Beyond numerical loss, sepsis rapidly remodels T cell composition and helper programs [9, 10, 22, 23]. Th polarization is dynamic over the early course of severe sepsis, making static labels biologically fragile [32]. Transcriptomic subclassification similarly delineates separable host-response states with distinct outcomes [26], and can align with differential treatment responsiveness (e.g., corticosteroids) [27]. Readouts such as CD4⁺/CD8⁺ ratio and Th bias are therefore most interpretable within an endotype and trajectory framework rather than as isolated markers.

### Markers of T cell exhaustion: checkpoint upregulation (PD-1, Tim-3, LAG-3)

A recurring sepsis phenotype is hyporesponsiveness with exhaustion-like features: rising inhibitory receptors alongside declining effector function. Early ICU immunophenotyping shows that patterns of exhaustion-marker expression on CD8⁺ T cells can stratify patients and associate with outcomes [33], and observational cohorts link PD-1/CTLA-4 expression to sepsis severity and prognosis [34]. Other checkpoints are implicated, including Tim-3 [35] and LAG-3; targeting LAG-3 can reverse T cell dysfunction and improve survival in murine polymicrobial sepsis [36]. Clinically, these associations motivate—but do not by themselves justify—checkpoint therapies. Checkpoint blockade has entered early clinical testing in sepsis (anti–PD-1 and anti–PD-L1), emphasizing feasibility, safety/tolerability, and pharmacodynamic immune effects in carefully selected settings [37, 38]. The modest signals observed to date are biologically plausible: sepsis-associated immunosuppression is rarely a simple excess of inhibitory signaling, but is frequently compounded by numerical depletion, immunometabolic exhaustion, and durable transcriptional/epigenetic rewiring of surviving T cells. In such contexts, releasing PD-1/PD-L1 “brakes” may be insufficient if the repertoire is already lost or functionally crippled, reinforcing the need for biomarker-enriched selection, timing sensitivity, and—potentially—combination strategies that also address cell number and metabolic fitness.

### Regulatory T cells in sepsis: protective, pathogenic, or context-dependent?

Tregs often expand or become proportionally enriched in sepsis, but directionality does not equal interpretation. In septic shock, increased circulating Tregs have been linked to lymphocyte anergy, supporting a role in adaptive suppression during part of the course [39]. However, in an exhaustion-marker–based ICU cohort, Treg levels were not clearly associated with outcome [33]. Clinically, the working model is conditional: Tregs may limit immunopathology during inflammatory surges, yet sustained regulatory dominance can entrench immunoparalysis in a subset of patients [39]. Apparent “Treg expansion” in blood can partly reflect proportional enrichment due to broad effector lymphocyte loss rather than true causal dominance. Therefore, Treg readouts should be interpreted alongside absolute counts, effector function, and trajectory (persistence vs recovery), and any Treg-targeted approach should rely on biomarker enrichment and longitudinal monitoring rather than static thresholds [10, 39].

### Decrease in B cells and dysfunction in antibody production

B cell injury is a core but under-measured dimension of sepsis immunopathology. Sepsis-induced apoptosis causes depletion of B cells in humans [28], and memory B cells appear particularly vulnerable, with activation-associated accelerated apoptosis described in critically ill septic patients [40]. Conceptual syntheses of B cell roles in sepsis outline therapeutic implications while emphasizing heterogeneity and the need for patient selection [41]. In practice, B cell phenotyping and functional humoral readouts are most informative as part of composite immune monitoring rather than as stand-alone predictors [40, 41]. Clinically, the most relevant downstream consequences include impaired humoral recall and antibody coordination during ICU recovery, which may predispose to secondary bacterial infections, delayed pathogen clearance, and vulnerability to viral reactivation in the immunosuppressed phase. These links are best interpreted longitudinally: persistence or failure to recover is typically more informative than early transient changes.

### Impaired cytotoxic function of NK cells

Sepsis also dampens innate cytotoxicity. Clinical studies report altered NK cell phenotype and reduced function in critically ill septic patients [42], and a shift toward inhibitory receptor dominance with impaired effector programs has been linked to sepsis-associated immunosuppression [43]. Together, these findings support a coordinated suppression of adaptive and innate cytotoxic arms during sepsis [42, 43]. Clinically, the most actionable downstream consequence is loss of cytotoxic containment, which can translate into impaired control of viral and intracellular pathogens and increased risk of secondary/opportunistic infections during convalescence. As with other immune domains, persistence and delayed recovery—rather than early transient suppression—best identify patients at risk.

### Immunosenescence meets sepsis: lymphocyte profile in elderly patients

#### Precondition: characteristics of immune aging at baseline

Older adults account for a disproportionate share of sepsis and sepsis-related deaths, reflecting biology as much as demographics [1, 7, 44]. In baseline immunosenescence, three constraints matter for sepsis: reduced naïve reserve, accumulated terminal differentiation, and limited reconstitution capacity. Thymic involution reduces de novo T cell output, contracts the naïve pool, and shifts maintenance toward antigen-experienced memory compartments—limiting repertoire diversity precisely when new clones are needed during severe infection [45, 46]. Chronic antigenic pressure further drives terminal differentiation; late-differentiated phenotypes (often CD28− and sometimes CD57+/KLRG1+) expand with age and proliferate poorly, producing less IL-2 and offering limited repertoire flexibility [45–50]. Latent cytomegalovirus (CMV) amplifies this architecture via “memory inflation” and can confound immunophenotyping unless serostatus is explicit [49, 51–53]. Humoral aging runs in parallel, with reduced B cell output and repertoire diversity blunting first-time responses to unfamiliar antigens [54, 55]. NK cell number may be preserved, but receptor remodeling and subset heterogeneity often translate into reduced cytotoxic and immunoregulatory capacity [56, 57]. Together, aging compresses lymphocyte breadth, lowers regenerative reserve, and narrows the ceiling for immune reconstitution under septic stress.

### Sepsis-induced exacerbation of immunosenescence

#### T cells

Sepsis delivers a systemic shock to lymphocyte biology—acute lymphopenia followed by functional paralysis among surviving cells [9, 25, 58]. In older adults, this insult lands on a smaller naïve reserve and a more terminally differentiated landscape, so the depth is often greater and the rebound weaker [45, 47, 48, 58]. The dominant problem is frequently failure of immune reconstitution rather than simple “delayed recovery.” Integrated evidence links persistent inflammation with exhaustion features and worse survival, underscoring that late outcomes track failed rebuilding as much as early inflammatory intensity [59].

This aged post-sepsis trajectory converges on three linked patterns: (i) durable loss of naïve T cells with constrained repertoire rebuilding [45–47, 52]; (ii) expansion of late-differentiated/senescent-like subsets (including CD28− phenotypes) that dominate the pool yet respond poorly to new antigens [48–50]; and (iii) more prominent exhaustion biology with dampened proliferative/cytokine programs and stronger inhibitory signaling in aged or repeated-sepsis models [12, 60]. Tregs add conditionality rather than a uniform direction: net effect depends on timing, tissue context, and depth of concurrent effector collapse—conditions older patients may reach earlier and sustain longer [9, 58, 61].

#### B cells

Older adults enter sepsis with reduced repertoire flexibility and weaker primary responses to unfamiliar antigens [54, 55]. Sepsis then further strips and distorts B cell compartments. In a prospective cohort of critically ill septic patients, memory B cells showed activation-associated accelerated apoptosis, offering a clinical mechanism for impaired humoral recall and vulnerability to secondary infection [60]. The expected downstream phenotype is prolonged susceptibility: fewer effective memory responses and poorer antibody coordination during ICU recovery [9, 40, 58, 60]. Clinical take-home: in older adults, memory B cell attrition and impaired humoral coordination are most consequential when they persist beyond the acute phase, where they plausibly contribute to secondary infections and delayed recovery.

#### NK cells

Sepsis perturbs NK cells at both numerical and functional levels, but the direction and consequence can vary by model, compartment, and phase of illness [62, 63]. Aging matters because baseline NK functional reserve is already reduced in many individuals [56, 57]. During sepsis, older patients may therefore experience a “double hit”: diminished baseline cytotoxic capacity plus slower functional recovery, potentially contributing to late viral reactivation or secondary bacterial/fungal infections when adaptive immunity is also compromised [7, 9, 56, 58, 62]. Clinical take-home: the NK signal is most clinically relevant in the late/post-acute phase, when persistent cytotoxic impairment aligns with viral reactivation risk in the setting of concurrent lymphopenia.

### Clinical implications

The key clinical feature in sepsis in older adults is failed immune reconstitution. Older patients more often transition from acute sepsis into prolonged immune dysfunction with recurrent infections and poor longer-term outcomes [7, 9, 58, 64]. Three practical implications follow. First, late death and secondary infections likely reflect persistent lymphopenia/exhaustion phenotypes as much as pathogen burden itself [25, 60, 65]. Second, standard care remains essential, but host-defense rebuilding becomes a distinct therapeutic problem—supporting longitudinal immune monitoring and endotype-aware adjunctive strategies in selected older patients [9, 58, 64]. Third, trials should treat age as a biology modifier by incorporating baseline repertoire constraints and key modifiers (e.g., CMV status and exhaustion signatures) that reshape risk and treatment responsiveness [51–53, 58, 59].

### Immunoimmaturity in the crucible: lymphocyte profile in pediatric sepsis

#### Precondition: the developing immune system at baseline

Children do not enter sepsis with a “scaled-down” adult immune system. They enter with an immune architecture that is actively assembling, recalibrating, and age-tuning its own thresholds. In neonates and young infants, adaptive immunity is dominated by naïve T and B cells; effector differentiation and polarization remain constrained, and early responses often lean toward Th2-skewed and tolerance-prone programs [66–68]. Large-scale immunophenotyping confirms that leukocyte composition and proportionality differ sharply between neonates and adults, making “normal ranges” inherently age-conditioned rather than transferable across life stages [69]. Neonatal CD4⁺ responses can also be functionally imbalanced, shaping the quality and durability of antimicrobial protection even when cell numbers appear reassuring [70].

Two additional baseline modifiers are easy to misread but clinically important. Humoral immunity is still learning to generalize: the infant B cell receptor repertoire expands rapidly with early-life exposures and is environmentally shaped, constraining responses to unfamiliar pathogens in the first months of life [71]. NK cells are phenotypically present early, yet functional capacity is reduced relative to later life, weakening baseline cytotoxic containment of viral and intracellular threats [72]. Maternal immunity adds a dynamic layer via transplacental IgG transport [73, 74], microbiome–driven IgG effects on neonatal ecology and immune tone [75], and breast milk antibodies that provide mucosal coverage while shaping immune development [76]. Breastfeeding also delivers bioactive signals that sculpt immune maturation and colonization patterns linked to immune training [77]. Finally, early-life CD8⁺ biology follows developmentally tuned programs rather than adult-like trajectories, which matters when “exhaustion-like” labels are applied to young hosts [78].

### Sepsis as a disruptor of immune maturation

#### T cells

Pediatric sepsis can trigger profound quantitative loss and qualitative T cell dysfunction on top of a system still building its effector and regulatory circuits. Two points guide interpretation: pediatric thresholds are development-dependent, and trajectories (persistence vs recovery) matter more than single snapshots. International pediatric sepsis guidelines acknowledge response heterogeneity and the incomplete translation of adult immunobiology to children [79]. PODIUM operationalizes immune injury as organ dysfunction by incorporating immune criteria (absolute lymphocyte count thresholds and age-adjusted CD4⁺ deficits) alongside innate competence signals such as low monocyte HLA-DR and impaired ex vivo TNF-α production capacity [80].

Prospective pediatric studies sharpen mechanism: early adaptive immunosuppression in septic shock includes reduced CD4⁺ cytokine-production capacity paired with lymphopenia, aligning with infection-related adverse outcomes [81]. Broader profiling links early innate and adaptive suppression to longer organ dysfunction duration, reinforcing that trajectories—direction, persistence, and recovery—carry more information than a single measurement [82]. Persistent lymphopenia associates with prolonged MODS and PICU mortality, again pointing to failure-to-rebound as a pragmatic bedside risk signal [83]. Function matters as much as counts: lymphocyte functional impairment and immunometabolic dysregulation vary by pathogen type, so pediatric sepsis cannot be reduced to one immunologic archetype [84]. Treg behavior in children likely reflects higher plasticity because immune set-points are still forming; clinically, interpret Treg signals against concurrent effector failure, pathogen burden, and recovery kinetics rather than as a universal “good” or “bad” marker.

#### B cells

Sepsis intersects with a humoral compartment that is still consolidating repertoire breadth and memory structure. Because early-life B cell diversity is constrained and dynamically shaped [71], sepsis-associated depletion or paralysis may impair immediate antibody-mediated defense and, in survivors, disrupt consolidation of durable immune memory. That possibility is clinically relevant in children who recover from the initial insult yet remain vulnerable to recurrent infections during convalescence. Clinical take-home: in pediatrics, B cell depletion/paralysis may compromise not only acute antibody defense but also consolidation of durable humoral memory, implying a longer follow-up horizon and recovery-oriented endpoints.

#### NK cells

NK cells may appear numerically “available” early in life, yet functional reserve is limited at baseline [72] and can be further dampened during severe systemic inflammation. In young children—particularly in viral or mixed infections—this can translate into impaired cytotoxic containment precisely when rapid control is most needed. Clinical take-home: when NK cytotoxic reserve remains suppressed alongside adaptive dysfunction, the downstream clinical signature may include viral persistence or reactivation and mixed secondary infections during the post-acute course.

#### Checkpoint and “exhaustion-like” states: neonatal and pediatric nuances

Checkpoint pathways are increasingly visible in neonatal sepsis biology. In septic newborns, higher PD-L1 expression on CD8⁺ lymphocytes has been linked to survival status, consistent with clinically observed differences in CD8⁺ abundance and inhibitory signaling between survivors and nonsurvivors [85]. Reviews of neonatal sepsis immunobiology place checkpoint proteins (including PD-1/PD-L1) within a plausible mechanistic framework for neonatal immunosuppression and as candidates for biomarker and therapeutic exploration, while still recognizing the current evidence gap for routine use [86]. Crucially, in neonates and young children, an “exhaustion-like” phenotype should not be assumed to be mechanistically equivalent to adult chronic antigen–driven T cell exhaustion. Developmentally programmed immune restraint—designed to calibrate inflammatory thresholds and limit immunopathology during tissue growth—can yield phenotypic similarity (e.g., higher inhibitory signaling) without implying maladaptive, fixed dysfunction. For this reason, we use the term “exhaustion-like” in pediatrics and interpret checkpoint readouts through timing and function. A pediatric checkpoint-dominant state is more concerning when it (i) persists beyond the early acute phase despite clinical stabilization and pathogen control, (ii) is accompanied by sustained functional impairment (e.g., reduced cytokine production, proliferation, or cytotoxic capacity on ex vivo assays), and (iii) aligns with downstream clinical signatures such as secondary infections or viral reactivation. Where these criteria are not met, elevated checkpoint signaling may reflect protective restraint rather than a therapeutic target. This checkpoint framing must be interpreted inside the neonatal innate immune context, where pattern-recognition signaling and its regulation differ from later life [87].

### Clinical Implications: rapid switching, endotype movement, and the risk of persistent immunosuppression

Pediatric sepsis often presents as coexisting—and sometimes rapidly alternating—hyperinflammation and immunosuppression rather than a tidy temporal handoff. Transcriptomic work in pediatric septic shock shows that endotype assignment can shift during the first days of illness, and these transitions track changing risk and treatment responsiveness; immune state is dynamic and early trajectories are potentially modifiable [88]. A Lancet Child & Adolescent Health Series analysis similarly centers developmental susceptibility and host-response differences as reasons pediatric sepsis demands age-attuned models rather than adult extrapolation [89].

For a subset of children, the aftermath is not an immunologic “reset” but a prolonged syndrome characterized by persistent inflammation, immunosuppression, and catabolism (pPICS), documented in sepsis-related mortalities and framed as clinically meaningful [90]. Pediatric perspectives consolidate PICS/pPICS as a lens for chronic critical illness in children, with lymphopenia and recurrent infections as recurring features [91]. These realities connect directly to how pediatric organ dysfunction is defined and monitored: PODIUM elevates immune dysfunction to organ dysfunction criteria, which should encourage serial, age-calibrated immune monitoring for trial design and bedside risk stratification strategies [80, 92].

### Head-to-head comparison: contrasts and unanticipated similarities

Sepsis is a syndrome, not an immunological monolith. Under the same clinical label, baseline age-shaped lymphocyte architecture determines what sepsis can deplete, what it can silence, and what can plausibly rebuild. The same bedside phenotype—lymphopenia followed by secondary infections—can therefore encode different biology and require different trial logic in older adults versus children [9, 10, 93]. To strengthen clinical interpretability, we time-anchor the comparison. During Day 0–1, many readouts reflect the acute host-response milieu and can be transient; interpretation depends on whether sampling is anchored to infection onset, sepsis recognition, or ICU admission. By Day 2–4, persistence (“failure to rebound”) of lymphopenia and delayed recovery of antigen-presenting capacity more strongly indicate sustained immunosuppression than early nadirs. By Day 4–7, persistent functional suppression despite stabilization and source control is more consistent with a deep immunoparalysis-like signature, whereas beyond one week, PICS/pPICS frameworks contextualize prolonged morbidity and recurrent infections.

**Fig. 3:**
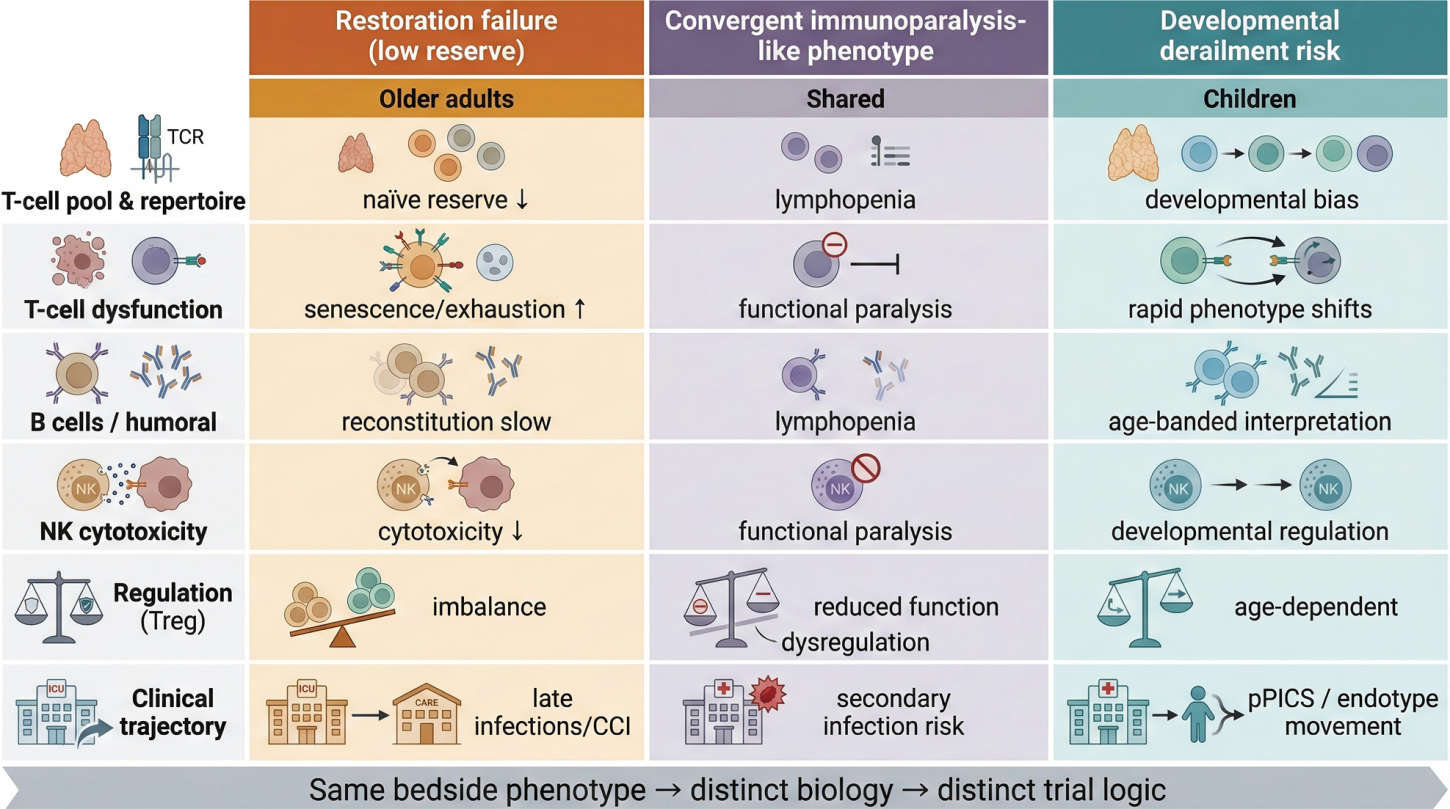
Head-to-head comparison of lymphocyte biology in elderly versus pediatric sepsis. Across extremes of age, severe sepsis can converge on profound lymphopenia and multi-lineage functional impairment, consistent with an immunoparalysis-like trajectory. However, the dominant constraint differs: in older adults, immunosenescence limits reserve and reconstitution, predisposing to restoration failure and persistent dysfunction; in children, sepsis can disrupt or misdirect developmental immune programs, producing rapidly shifting phenotypes that do not map cleanly onto adult exhaustion frameworks. This synthesis highlights why the same bedside phenotype may represent distinct biology, motivating age-stratified interpretation of lymphocyte subsets and endotype-aware trial designs.Time-anchored interpretation is essential: early nadirs are often shared across ages, whereas persistence (“failure to rebound”) during Day 2–4 and sustained dysfunction by Day 4–7 are more informative for identifying true recovery failure and trial-enrichable phenotypes. Translational take-home: the same bedside phenotype should trigger different biomarker panels, timing windows, and adjunct strategies in older adults versus children; age-agnostic enrollment risks diluting treatment signals.

Building on the elderly- and pediatric-focused sections above, Table 2 synthesizes quantitative and qualitative changes across lymphocyte subsets, and Fig. 3 summarizes convergent and divergent pathways (“restoration failure” versus “developmental derailment”) to guide age-stratified trial logic.

**Table 2:** Sepsis-induced lymphocyte subset alterations: contrasts and shared pathways in elderly vs pediatric patients.

Axis/subset	Shared sepsis effect (common pathway)	Elderly—age-amplified pattern	Pediatric—developmental nuance	Representative evidence
Lymphopenia (ALC kinetics)	Lymphopenia is common; persistence (failure to rebound) aligns with mortality/secondary infection risk [24, 25, 107, 121]	Deeper hit + weaker rebound due to low naïve reserve and limited regenerative capacity [45–49, 59, 60]	Early/persistent lymphopenia associates with prolonged MODS/mortality; thresholds must be age-adjusted [80, 83]	Adult: persistent lymphopenia predicts mortality [25]; Pediatric: early persistent lymphopenia risk [83]
CD4⁺ T cells	Apoptosis-driven depletion and functional paralysis reported in human sepsis [12, 28]	Reduced proliferative capacity; exhaustion-like signatures may persist longer [59, 60]	Reduced CD4 cytokine-production capacity documented early; may shift rapidly with time/treatment [81, 82, 88]	Autopsy/human apoptosis [12, 28]; Pediatric adaptive suppression [81, 82]
CD8⁺ T cells	Exhaustion-marker upregulation (PD-1/Tim-3/LAG-3) and hyporesponsiveness in subsets [33–36]	More senescent/terminal phenotypes dominate post-sepsis pool (e.g., CD28⁻) and respond poorly to new antigen demands [46, 50, 51, 60]	“Exhaustion-like” interpretations must consider developmental programs; neonatal checkpoint signals reported [78, 85, 86]	ICU exhaustion patterns [33]; neonatal PD-L1 signal [85]
Th polarization/CD4:CD8 ratio	Dynamic shifts; single time-point labels unreliable [32]	Bias toward limited repertoire flexibility and delayed immune recovery [46, 94–97]	Developmental Th2 bias/tolerance-prone responses in early life; endotype transitions common [66–70, 88]	Dynamic Th1/Th2 changes [32]; pediatric endotype transition [88]
Regulatory T cells (Treg)	Often proportionally enriched; effect depends on timing and competing effector collapse [39]	Regulatory dominance may entrench immunoparalysis when effector compartment collapses early/persistently [45, 61]	Higher plasticity but interpretation must be trajectory-based, not “good/bad” [79–82]	Treg contribution to anergy in septic shock [39]
B cells/humoral function	B cell apoptosis and memory B cell vulnerability described [28, 40, 41]	Baseline repertoire narrowing + sepsis depletion → impaired recall and secondary infection vulnerability [55, 56, 60]	Humoral system still learning; sepsis may disrupt consolidation of durable memory [71]	Human depletion [28]; memory B cell accelerated apoptosis [40]
NK cells	Phenotype/function impaired; inhibitory receptor dominance described [42, 43]	Baseline cytotoxic decline + slower recovery (“double hit”) [57, 58, 62]	Functional reserve limited at baseline; may be especially relevant in viral/mixed infections [72]	NK phenotype/function in sepsis [42, 43]; newborn NK function reduced [72]
Clinical endpoint convergence	Both can progress to immunoparalysis-like state with late infections; PICS/CCI or pPICS framework captures prolonged morbidity in subsets [90, 91, 101, 102]	More likely “failure to reconstitute” and late death/readmission trajectory[7, 64, 119]	“Rapid switching/endotype movement”; pPICS increasingly recognized in pediatric critical illness [88–91]	PODIUM and pPICS perspectives [80, 90, 91]

“Shared pathways” indicate concordant directional changes during sepsis, whereas magnitude, timing, and mechanisms may diverge between older adults and children. Findings are sensitive to time anchoring (infection onset vs sepsis recognition vs ICU admission), single versus serial sampling, and platform/gating differences; whenever feasible, interpret ALC and subset changes as trajectories (persistence/failure to rebound) rather than single cutoffs. Pediatric interpretation requires age-adjusted reference intervals due to rapid developmental shifts in lymphocyte composition. Clinical take-home: interpret each lymphocyte subset by time window (Day 0–1 vs persistence) and age-calibrated baseline, because similar directions of change can encode different mechanisms and recovery ceilings.

*ALC* absolute lymphocyte count, *MODS* multiple organ dysfunction syndrome, *NK* natural killer, Treg regulatory T cell.

### Contrasts

#### Basic state: aging’s “exhaustion” vs development’s “immaturity”

Older adults enter sepsis with a lymphocyte compartment already constrained by thymic involution, reduced naïve T cell availability, cumulative antigen exposure, and limited repertoire renewal. Reserve is lower, reconstitution is slower, and the margin for recovery after a systemic hit is narrow [45, 48, 94]. Children enter sepsis while adaptive immunity is still being assembled. Effector control and restraint are actively being calibrated, inflammatory thresholds are age-tuned, specialization remains incomplete, and humoral competence is still maturing [14, 95].

#### T cell pool: reduced diversity vs diversity still being built

In older adults, TCR diversity contracts in a subset-specific pattern, with particularly marked attrition in naïve CD8⁺ pools and compensatory expansion of memory clones. The repertoire becomes less flexible, shrinking the “solution space” for responses to new pathogens [96, 97]. Persistent CMV exposure can further remodel the T cell pool and is often discussed as an accelerator of immune-aging signatures, adding inter-individual heterogeneity to an already constrained baseline [98]. In children, repertoire breadth is not the product of decades of immune history; it is still being established. Neonatal and infant T cells can display developmental functional biases that favor rapid, innate-like programs—sometimes protective, sometimes restrictive when classical effector differentiation is needed—depending on the inflammatory milieu [95, 99].

#### Main defect focus: failure to restore vs derailment of a program

In older adults, the dominant limitation after sepsis is often the inability to reconstitute effective adaptive immunity once it collapses: a prolonged “low-reserve, low-recovery” state in which exhaustion-like features and functional impairment can persist [60]. In children, the central risk is not only suppression but misdirection: sepsis can disrupt the normal trajectory of immune maturation, generating phenotypes that do not map cleanly onto adult exhaustion frameworks and can shift rapidly with time and treatment [14, 95]. Moreover, some checkpoint-dominant patterns in early life may reflect developmentally programmed restraint rather than intrinsically maladaptive failure, making functional confirmation and trajectory persistence essential before labeling pediatric T cells as “exhausted”.

#### Recovery potential: higher plasticity in children

Children retain stronger regenerative capacity, including higher thymic activity across early life, which can support re-expansion and re-balancing of lymphocyte subsets once the insult is controlled [14, 45]. Immunorestoration in older adults is intrinsically less efficient: baseline maintenance relies more on peripheral self-renewal than de novo thymic output, leaving less reserve to compensate after profound depletion [45, 94].

### Potential similarities

#### Severe sepsis can drive profound lymphopenia and downstream dysfunction at both extremes of age

Across age groups, severe sepsis can converge on deep lymphopenia and downstream vulnerability to secondary infection and late mortality [10, 25]. However, the time dimension is critical: profound lymphopenia during Day 0–1 may reflect acute inflammatory and neuroendocrine stress and can be transient, whereas persistence (“failure to rebound”) by Day 2–4 is a more actionable signal of sustained immune dysfunction. In adults, persistent lymphopenia after sepsis diagnosis associates with adverse outcomes and has been proposed as a clinically accessible marker of sepsis-associated immunosuppression [25]. In children, early and/or persistent lymphopenia and broader adaptive suppression similarly track worse trajectories and prolonged organ dysfunction [81–83], but interpretation must be age-calibrated and anchored to developmental baselines and rapid endotype movement during the acute phase.

#### Treg cells: a key but clinically ambiguous regulator

Treg expansion or relative predominance after septic shock has been linked to reduced lymphocyte proliferative capacity, supporting a plausible role in post-sepsis “anergy-like” states [39]. The same directional change can also be protective when inflammation is uncontrolled because Tregs can limit immunopathology and tissue injury. Interpretation therefore hinges on timing, pathogen control, and competing immune deficits, not on Treg counts alone [10, 39].

#### Both extremes struggle with “new” pathogens—but for different reasons

Older adults may fail because repertoire attrition and reduced naïve reserve constrain the generation of new antigen-specific responses, particularly in cytotoxic compartments [96, 97]. Children may fail because durable, high-quality memory and class-switched humoral responses are still under construction, and early-life functional biases can reshape the quality and durability of adaptive programming after an extreme inflammatory event [14, 95, 99].

#### Both can enter an immunoparalysis-like state with high risk of secondary infections

In pediatric critical illness, immunoparalysis phenotypes (often operationalized with functional/biomarker frameworks rather than single markers) have been associated with nosocomial infection risk and adverse outcomes [82, 100]. In adults, immunosuppression is strongly implicated in late complications and death, supported by tissue-level immunopathology in fatal sepsis and by clinical patterns of secondary/opportunistic infections [10, 12]. At the syndromic level, persistent inflammation coupled to immunosuppression and catabolism (“PICS”/chronic critical illness) provides a shared endpoint where recurrent infection, poor recovery, and prolonged ICU trajectories cluster—though the routes into that state may differ by age [101, 102].

Importantly, early Day 0–1 “snapshot” depressions should not be labeled immunoparalysis; the term is most defensible when functional suppression persists despite stabilization and source control (often evident by Day 4–7) and aligns with ongoing nosocomial infection risk. A useful comparison is not “elderly versus children,” but “low-reserve restoration failure” versus “developmental derailment.” Precision trials should therefore (i) stratify by immune trajectory rather than admission snapshots, and (ii) use endotype-aware designs—well illustrated by pediatric transcriptomic subclassification and endotype transition work—while explicitly accounting for baseline immunological constraints in older adults [60, 88, 94, 103].

### Translational perspectives and future directions

#### Biomarkers for age-specific immunomonitoring

Sepsis immunobiology is heterogeneous and time-dependent; single “snapshot” measurements rarely translate into defensible treatment decisions [9, 10, 104, 105]. Two practical questions guide biomarker use: what is the age-calibrated baseline, and does the defect persist (“failure to rebound”) after initial stabilization? Biomarker strategies should therefore prioritize trajectory (direction and persistence) and function, using age-calibrated panels rather than isolated thresholds [104–106].

In older adults, a pragmatic approach combines global adaptive reserve (serial absolute lymphocyte count, ALC), innate antigen-presenting competence (monocyte HLA-DR, mHLA-DR), and adaptive exhaustion/senescence readouts (e.g., PD-1 on T cells; senescent CD28⁻/CD57⁺ expansions) [24, 48, 60, 94, 107–110]. The key signal is failure to rebound: persistent lymphopenia aligns with secondary infections and mortality more consistently than the initial nadir [24, 107]. For mHLA-DR, interpret risk through persistence and recovery kinetics rather than transient early depression; translation depends on assay standardization and time-anchored interpretation [108–110].

**Fig. 4:**
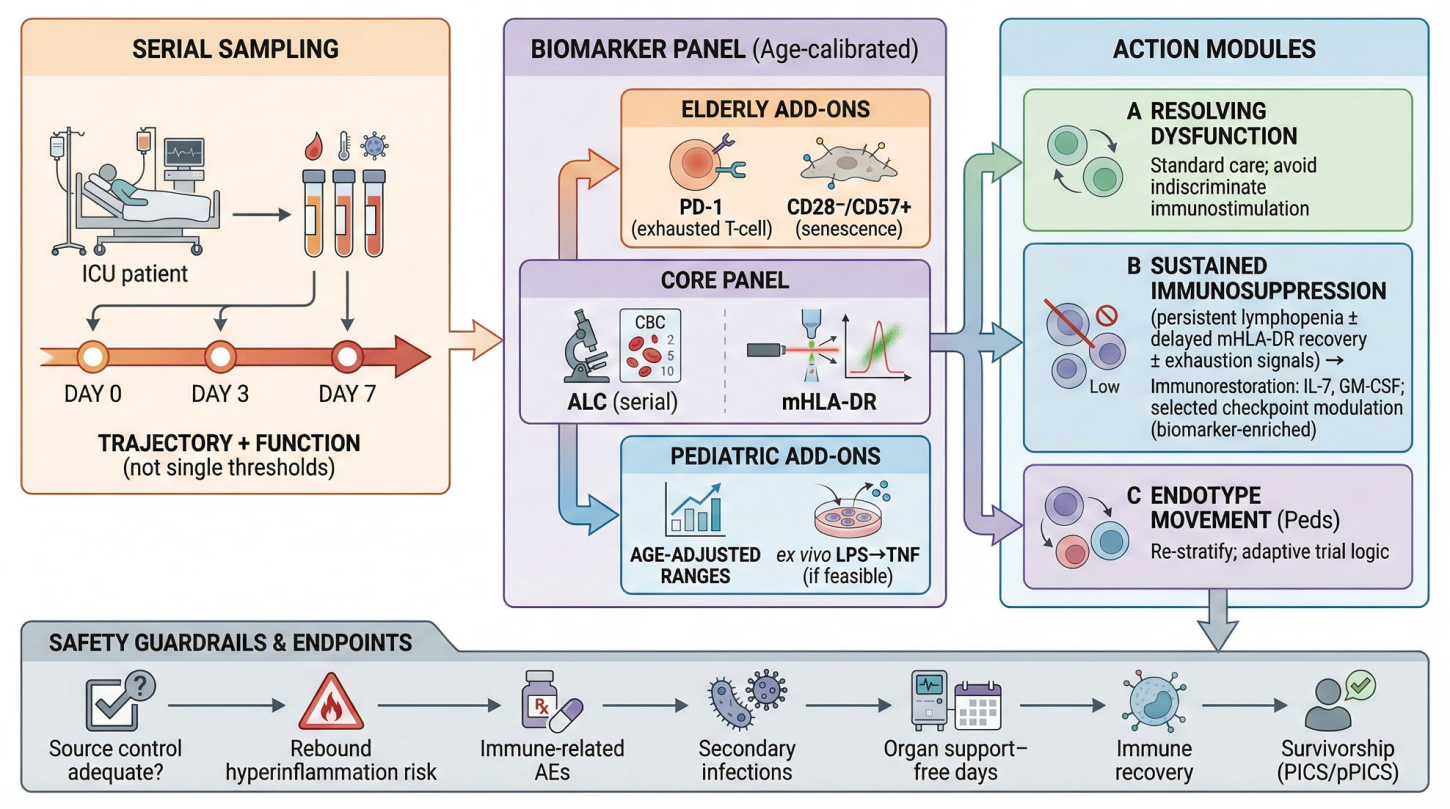
Biomarker-informed bedside framework to operationalize age-stratified precision immunology in sepsis. Because sepsis immunobiology evolves over time, actionable decisions require serial, age-calibrated monitoring that integrates trajectory (direction and persistence of change) with functional capacity. A pragmatic core panel pairs global immune reserve (serial absolute lymphocyte count, ALC) with antigen-presenting competence (monocyte HLA-DR, mHLA-DR), supplemented in older adults by exhaustion/senescence signals (for example, PD-1 expression and CD28⁻/CD57⁺ expansions) and in children by age-adjusted thresholds and feasible functional readouts (for example, ex vivo LPS-induced TNF production). Patients demonstrating sustained immunosuppression after initial resuscitation may be candidates for biomarker-enriched immunorestorative strategies, whereas resolving dysfunction supports avoidance of indiscriminate immunostimulation. Safety guardrails and clinically meaningful endpoints (secondary infections, organ support–free days, immune recovery, and longer-horizon survivorship) are essential for translation. Translational take-home: treat immunoadjuvant therapy as a trajectory-gated intervention—support immune reconstruction when defects persist, and avoid indiscriminate immunostimulation when trajectories are resolving.

In children, “normal” immunity is developmental and strongly age-stratified. Panels integrating ALC, mHLA-DR, and—when feasible—ex vivo functional assays (e.g., LPS-induced TNF production) align with pediatric immune dysfunction constructs and help separate transient suppression from clinically relevant immunoparalysis [79–81, 111]. Because pediatric endotypes can shift during the acute phase, serial profiling is particularly valuable for re-stratifying risk after initial stabilization [88]. To operationalize this logic, Table 3 links age-calibrated biomarker panels to best-fit therapies and safety guardrails, and Fig. 4 frames a bedside workflow for serial re-stratification.

**Table 3:** Biomarker-informed, age-stratified immunomonitoring and immunoadjuvant therapy.

Decision time window	Minimal “trial-ready” immune panel	Enriched phenotype to flag	Candidate adjunct strategy	Safety guardrails & endpoints	Age-specific notes
Day 0–1					
(baseline + early trajectory)	ALC (serial), mHLA-DR (baseline), +/− CD4/CD8 counts	Early profound lymphopenia plus low antigen-presentation reserve (low mHLA-DR) suggests high risk for sustained immune dysfunction	Do not reflexively immunostimulate; prioritize source control, avoid indiscriminate anti-inflammatory escalation; plan re-check at Day 2–3	Avoid “overshoot” inflammation; define infection-control endpoints; repeat sampling	Pediatric thresholds must be age-adjusted; immune states can shift early (re-stratification essential)
Day 2–4					
(persistence check/“failure to rebound”)	ALC kinetics (persistence), mHLA-DR recovery vs sustained suppression; +/− T cell checkpoint markers (PD-1), senescence markers (CD28⁻/CD57⁺)	Sustained lymphopenia and/or delayed mHLA-DR recovery = enriched “sustained immunosuppression” pattern	IL-7 as lymphocyte-restorative (proof-of-mechanism) [29]; consider GM-CSF in low mHLA-DR phenotype [112, 113]	Biomarker-enriched enrollment; monitor inflammatory rebound; endpoints beyond 28-day mortality (secondary infections, ventilator-free days, immune recovery metrics)	Elderly more likely persistent failure-to-reconstitute; pediatric more conservative, blood-volume constraints, developmental safety
Day 4–7					
(deep immunoparalysis signature)	ALC + mHLA-DR + functional assay if feasible (ex vivo LPS → TNF)	Persistent immunoparalysis despite stabilization and source control	Checkpoint inhibition explored in early-phase sepsis trials (anti–PD-1/anti–PD-L1) [37, 38]; consider as selected and controlled strategy	Tight inclusion criteria; immune-related adverse events surveillance; infection vs inflammation balance	Pediatric: emphasize preservation of developmental programs; only in highly enriched settings (conceptual, not routine)
Beyond 1 week					
(chronic critical illness/PICS lens)	Longitudinal panel + clinical trajectory (recurrent infections, catabolism)	Prolonged immune dysfunction with recurrent infections/poor recovery	Multi-modal supportive strategy; trial endpoints should include long-horizon functional outcomes	Functional outcomes, readmissions, late infections	Elderly: frailty/comorbidities confound; pediatrics: pPICS framing and survivorship outcomes matter

This table provides a translational framework linking age-calibrated immune monitoring to trial-enrichment logic and time-sensitive deployment of immunoadjuvant strategies, directly addressing common drivers of negative sepsis trials (age-agnostic enrollment, inadequate immune phenotyping, lack of trajectory confirmation, and insensitive endpoints); it is not a standalone clinical guideline. “Day 0” should be defined (sepsis recognition or ICU admission), and sustained immunosuppression should be inferred from repeated measurements (e.g., persistent lymphopenia and/or delayed mHLA-DR recovery) rather than single thresholds. Candidate immunotherapies should be considered only with biomarker-enriched selection and predefined safety guardrails to mitigate rebound hyperinflammation and infection-control failure. Translational take-home:Table 3 operationalizes trajectory-gated enrollment—use serial confirmation of persistence to match patients to adjunct strategies and endpoints, thereby reducing the risk of null trials from immune-state misclassification.

*ALC* absolute lymphocyte count, *mHLA-DR* monocyte human leukocyte antigen–DR, *LPS* lipopolysaccharide, *TNF-α* tumor necrosis factor alpha, *GM-CSF* granulocyte–macrophage colony-stimulating factor, *IL-7* interleukin-7, *ICU* intensive care unit, *VFD* ventilator-free days, *PICS* persistent inflammation, immunosuppression and catabolism syndrome, *CCI* chronic critical illness, *pPICS* pediatric PICS.

### Immunoadjuvant therapy

Historical disappointments in sepsis immunotherapy are increasingly interpretable as design failures rather than proof that immunoadjuvant strategies are futile. Many trials enrolled patients by syndrome label and calendar time (often anchored to ICU admission), with limited age stratification and inadequate immune phenotyping—thereby mixing transient early suppression with true recovery failure and diluting treatment signals. Mortality-only endpoints can also miss effects that would more plausibly appear as reduced secondary infections, improved immune recovery, or longer-horizon functional outcomes.

Accordingly, immunoadjuvant trials should be age-stratified and trajectory-based, requiring serial confirmation of persistence (“failure to rebound”) and phenotype-gated enrollment. Table 3 operationalizes this logic across decision windows (Day 0–1, Day 2–4, Day 4–7, and beyond 1 week), linking minimal trial-ready immune panels to enriched phenotypes, candidate adjunct strategies, and safety guardrails/endpoints.

In older adults, immunoadjuvant therapy should target a defined reconstitution-failure state, not early uncontrolled inflammation—signaled by persistent lymphopenia, low mHLA-DR, and exhaustion signatures [10, 106]. IL-7 has human trial evidence for lymphocyte restoration and remains a leading candidate for biomarker-enriched immune reconstruction strategies [29]. Checkpoint blockade has reached early-phase sepsis trials (anti–PD-1 and anti–PD-L1), establishing feasibility and pharmacodynamic engagement in carefully selected patients rather than supporting broad use [37, 38]. Myeloid-directed immunostimulation (e.g., GM-CSF) similarly argues for phenotype-gated deployment with dynamic monitoring to limit rebound inflammation [112, 113]. Preclinical work suggests IL-15 may amplify cellular immunity in aged settings, but remains hypothesis-generating without robust clinical validation [61].

In pediatrics, the goal is not indiscriminate “immune boosting,” but preventing infection-related immune collapse while preserving developmental programs [79, 80, 111]. Trials should enroll children with sustained immunoparalysis despite source control and hemodynamic stabilization, embed child-specific safety guardrails, and use endpoints that reflect both near-term infection control and longer-horizon functional outcomes [79, 80, 114].

### Challenges in pediatric and geriatric sepsis trials

Definitions and heterogeneity. Pediatric sepsis definitions and severity criteria continue to evolve, complicating cross-study comparability and reinforcing the need for immune-phenotype enrichment rather than syndrome-only enrollment [92, 115]. Dynamic subphenotypes can shift early in both adults and children, so enrollment-time labels may be insufficient; adaptive re-classification strategies are increasingly relevant [105, 106].

Ethics and feasibility. Pediatric trials face constraints in consent, blood volume, and acceptable risk while still requiring mechanistically meaningful immune endpoints [79, 80]. In older adults, multimorbidity, polypharmacy, competing risks, and baseline vulnerability (e.g., frailty) complicate both safety attribution and efficacy detection, pushing trial design toward geriatric-relevant stratifiers and patient-centered outcomes [116–118].

Endpoints beyond 28-day mortality. Mortality alone is often insensitive for immune-targeted interventions. Adult survivorship frequently includes durable cognitive/physical morbidity and readmissions, supporting longer-horizon recovery outcomes [119]. Pediatric survivorship similarly demands functional trajectories and post-sepsis morbidity frameworks to judge immunomodulatory trade-offs in a developing host [114, 120].

### Key unanswered questions

#### What are the age-stratified immune trajectories that truly predict “failure to recover”?

Large longitudinal cohorts with harmonized immune assays are needed to define trajectories (not just levels) that identify sustained immunosuppression vs resolving dysfunction across ages [105, 106, 108–110].

#### How should we operationalize immune endotypes for bedside use?

Consensus frameworks are emerging to unify terminology for sepsis-associated immune dysfunction (including immunosuppression/immunoparalysis) across studies, but translation requires reproducible biomarker panels, decision thresholds, and evidence that re-stratification changes outcomes [104–106].

#### Does early-life sepsis reprogram long-term immune health?

Beyond acute mortality, pediatric sepsis may derail developmental immune trajectories, with possible downstream effects on infection susceptibility, vaccine responses, and functional outcomes—questions that demand prospective follow-up embedded in acute-care studies [114].

## Conclusion

### Baseline immune state defines the “starting line”

Sepsis is best framed as systems-level immune failure in which inflammatory injury and immunosuppression often coexist rather than unfold as clean sequential phases [8, 9]. In the earliest hours, this syndrome is largely initiated and propagated by innate immune sensing and effector pathways; adaptive immune alterations frequently reflect the downstream cost of this acute host-response program. Within this framework, age is a biological prior that reshapes the lymphocyte compartment before infection and constrains what “recovery” can plausibly look like [10, 12]. Lymphopenia is the most visible footprint of adaptive immune derailment, mechanistically linked to apoptosis, impaired lymphopoiesis, and checkpoint-driven dysfunction [24, 25]. Clinically, persistence—failure to rebound—tracks ICU-acquired infection and death more consistently than the initial nadir, supporting serial lymphocyte monitoring as a pragmatic marker of sustained immune dysfunction in adults [107, 121].

### Convergent “immunoparalysis,” divergent biology

Both extremes of age can converge on an immunoparalysis-like phenotype, yet the routes and ceilings for recovery differ. Clinically, this means that the same syndrome label and even the same immune readout can demand different interpretation and different trial logic across ages; convergence in phenotype should not be mistaken for equivalence in mechanism or recoverability. In older adults, immunosenescence compresses the naïve T cell pool and expands terminally differentiated phenotypes, narrowing repertoire breadth and lowering the capacity for adaptive reconstitution after septic injury [14, 48, 94]. In children, the defining feature is developmental plasticity—an advantage that sepsis can distort, accelerate, or arrest, with potential consequences beyond the index hospitalization [14, 122]. Pediatric critical illness frameworks increasingly operationalize immune dysfunction as measurable and clinically relevant, while emphasizing that thresholds and trajectories must remain development-aware [80, 111]. Clinical data support early adaptive immunosuppression in septic children that associates with subsequent organ dysfunction, highlighting that pediatric immunoparalysis-like states (typically defined by longitudinal and functional frameworks) can be tightly coupled to the early host-response program [81, 82]. Transcriptomic studies further show that pediatric immune endotypes can transition rapidly, reinforcing the need for serial re-stratification rather than one-time labels [83, 123].

### Age-stratified precision immunology as the translational path

Translation requires immune monitoring that is biologically meaningful and operationally reproducible—one reason standardization of monocyte HLA-DR measurement has become central to trial readiness [110, 124]. Trial design is also shifting toward biomarker-guided and predictive enrichment as prerequisites for detecting treatment signals in a heterogeneous syndrome [106, 125]. A key lesson from prior failures is that enrolling by syndrome label without age calibration and immune-trajectory confirmation conflates biologically distinct states— including transient early suppression versus true recovery failure—and biases trials toward null results. The age-stratified, time-anchored framework (Table 3) provides a practical pathway to align enrollment, timing, endpoints, and safety guardrails with the specific immune defect being targeted. Early immunoadjuvant trials illustrate both promise and constraint: IL-7 can restore lymphocyte numbers in septic shock, and checkpoint blockade has shown feasibility in carefully controlled early-phase studies, underscoring that timing, selection, and safety guardrails are the core design problem [29, 37]. Similarly, anti–PD-L1 and GM-CSF experiences argue that immunostimulation is feasible but must be deployed against clearly defined immune defects, with longitudinal monitoring to prevent harm [38, 112]. The GRID trial highlights practical barriers—recruitment, phenotype stability, and endpoint sensitivity—while systematic reviews of GM-CSF reinforce that immune enhancement is a strategy class rather than a single-drug narrative [113, 126].
